# Supplementary material for: Elucidation of multiple high-resolution states of human MutSβ by cryo-EM reveals interplay between ATP/ADP binding and heteroduplex DNA recognition
Source: Nucleic Acids Res. 2025 Jul 4;53(12):gkaf604. doi: 10.1093/nar/gkaf604 (PMC12231599; doi:10.1093/nar/gkaf604)
Supplement: gkaf604_Supplemental_Files [file gkaf604_supplemental_files.zip › Lee_et_al_NAR_Revised_Supplementary_Data.pdf]

Supplementary data

Elucidation of multiple high-resolution states of human MutSβ by cryo-EM reveals interplay between ATP/ADP binding and heteroduplex DNA recognition

Jung-Hoon Lee[1,†], Maren Thomsen[1,†], Herwin Daub[1], Gabriel Thieulin-Pardo[1], Stefan C. Steinbacher[1], Agnieszka Sztyler[1], Vinay Dahiya[1], Tobias Neudegger[1], Celia Dominguez[2], Ravi R. Iyer[2], Hilary A. Wilkinson[2], Edith Monteagudo[2], Nikolay V. Plotnikov[2], Dan P. Felsenfeld[2], Tasir S. Haque[2], Michael Finley[2], Julien Boudet[2], Thomas F. Vogt[2], and Brinda C. Prasad[2,*]

[1]Proteros biostructures GmbH, Bunsenstr 7a, 82152 Martinsried, Germany

[2]CHDI Management/CHDI Foundation, Princeton, NJ 08540, USA

[†]The first two authors should be regarded as Joint First Authors.

[*]To whom correspondence should be addressed. Email: brinda.prasad@chdifoundation.org

Present address: Tobias Neudegger, Roche Diagnostics GmbH, Nonnenwald 2, 82377 Penzberg, Germany

Present address: Ravi R. Iyer, Script Biosciences, 2929 Arch Street, Philadelphia, PA 19104, USA

Present address: Nikolay V. Plotnikov, Treeline Biosciences, 11180 Roselle St, San Diego, CA 92121, USA

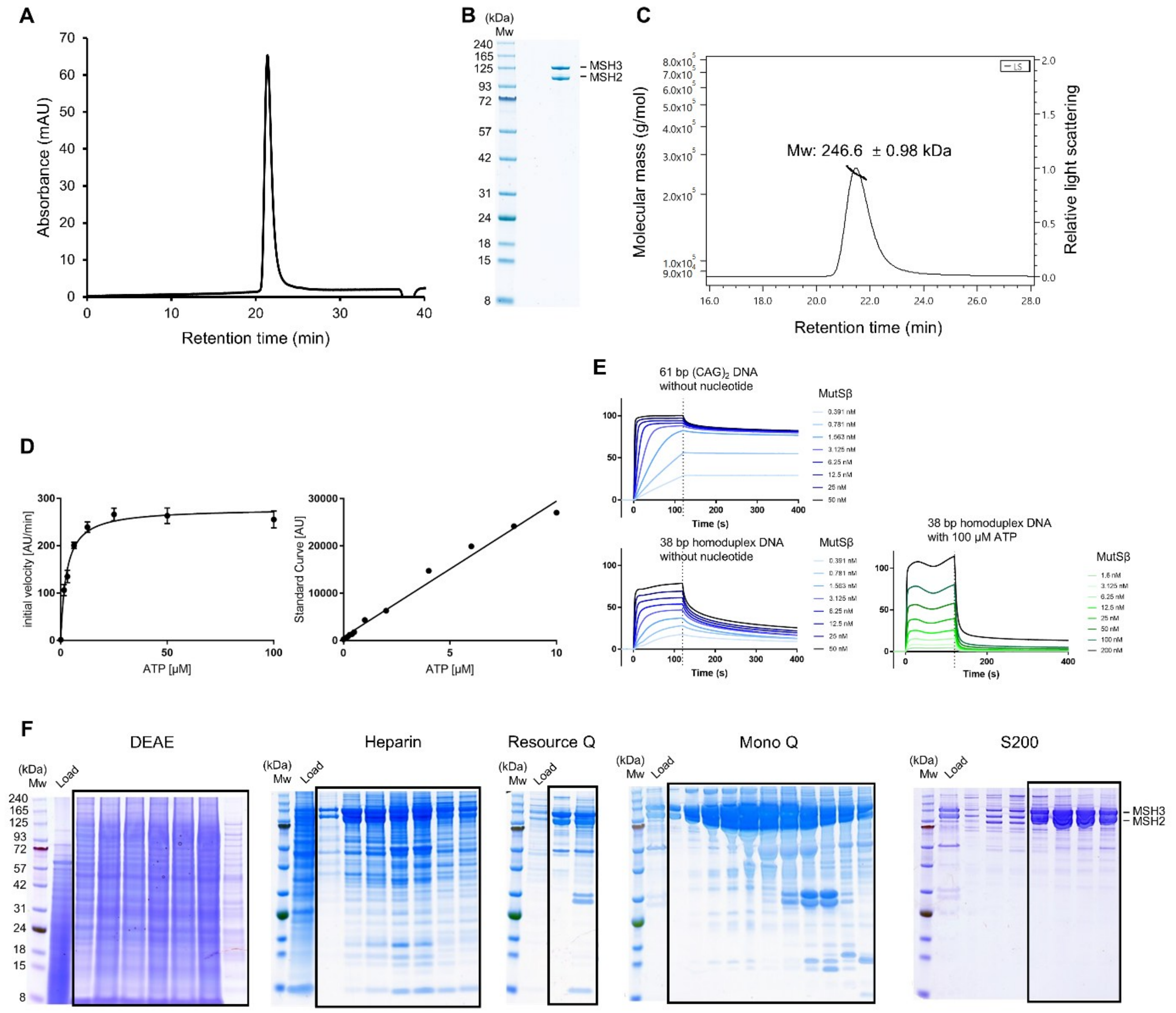

Supplementary Figure S1. Biochemical analysis of full-length human MutSβ. (**A**) SEC analysis of purified full-length MutSβ. (**B**) SDS-PAGE of purified MutSβ for cryo-EM grid preparation. (**C**) SEC-MALS analysis of purified full-length MutSβ. (**D**) ATPase activity determination of MutSβ by ADPglo in absence of DNA. Background subtracted initial velocity of ATP dilution series is plotted over ATP concentration (left). Non-linear fit of reaction time yields a V_{max} of 279 AU/min or 0.1 µM/min, converted using a slope of 2,872 AU/[µM] from linear regression (right; V_{max} = 0.12 µM/min; K_M = 6.9 µM; k_{cat} = 1.22 min^{-1}). (**E**) DNA binding of MutSβ to immobilized DNA measured by SPR. Affinity determination of MutSβ for homoduplex DNA yielded KDeq = 3.32 nM in the absence of ATP, while the ATP-bound form of MutSβ exhibited a 31-fold reduction in DNA affinity (KDeq = 102 nM). When a $(CAG)_2$ insertion loop was introduced, MutSβ's affinity drastically increased to KDkin = 67.2 pM, with kinetic rate constants of ka = 1.29 × 10^7 $M^{-1}s^{-1}$ and kd = 8.65 × 10^{-4} s^{-1}. As equilibrium was not reached under these conditions, the kinetic dissociation constant (KDkin) derived from the rate constants (ka and kd) was prioritized over the fitted equilibrium value (KDeq = 265 pM). (**F**) SDS-PAGE gel images of each purification step of full-length MutSβ. Pooled fractions used for subsequent purification steps are highlighted with black boxes.

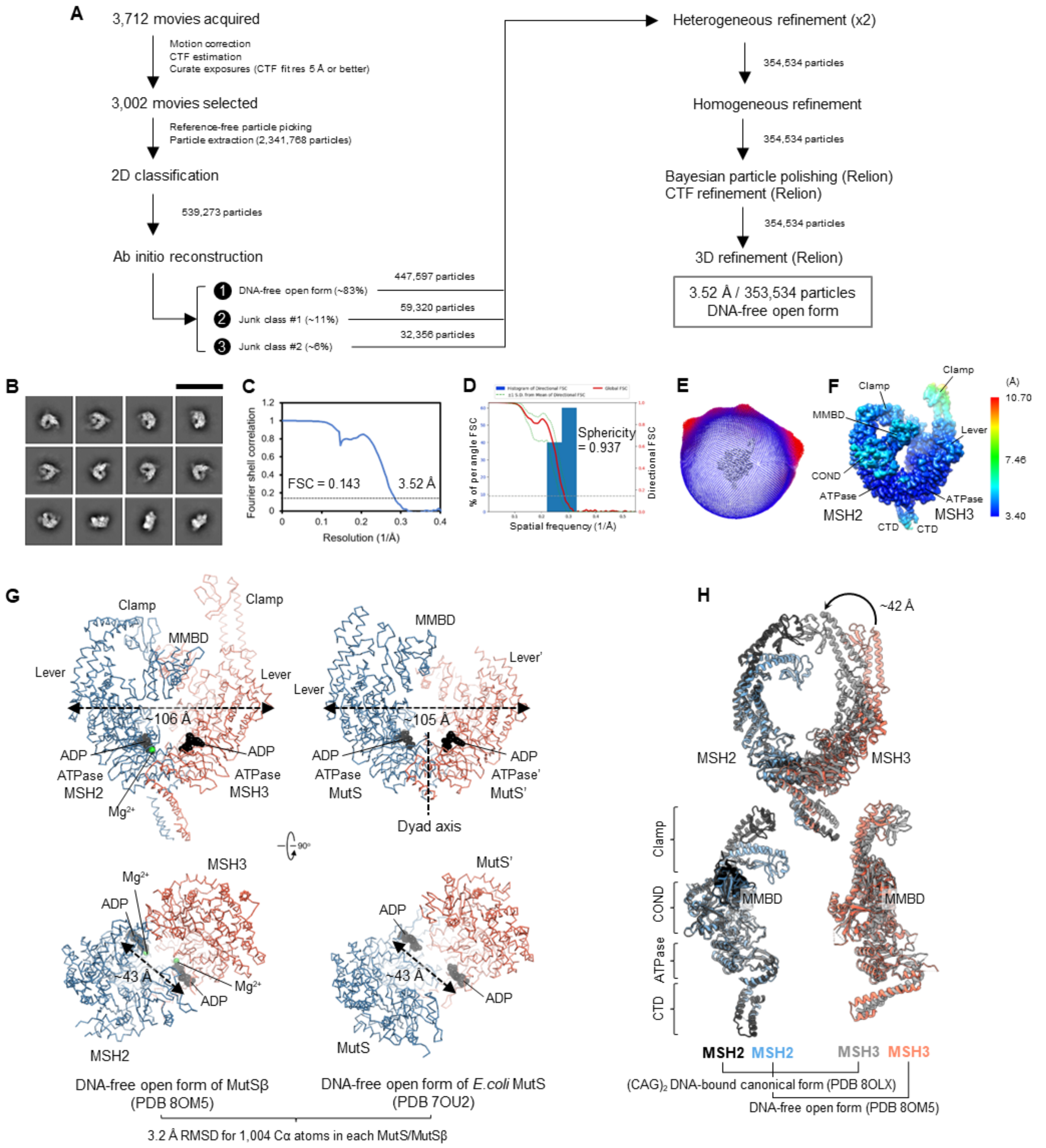

Supplementary Figure S2. Cryo-EM analysis of DNA-free open form of MutSβ-ADP complex (PDB 8OM5). (**A**) Summary of the image processing workflow. (**B**) 2D classes of DNA-free open form of MutSβ. The scale bar in white indicates 293 Å. (**C**) Gold-standard FSC curve for the density map of DNA-free open form of MutSβ. (**D**) 3D FSC plot for the density map of DNA-free open form of MutSβ. (**E**) Heat map showing particle orientation distribution. (**F**) Local resolution represented by a heat map on the density contour. (**G**) Ribbon diagrams of human MutSβ and *E. coli* MutS (PDB 7OU2) are shown in their DNA-free open form, based on superposition. (**H**) Relative domain movements of the open conformation of MutSβ compared to the canonical mismatch-bound form (PDB 3THZ) (DNA, MMBD and connector are omitted for clarity).

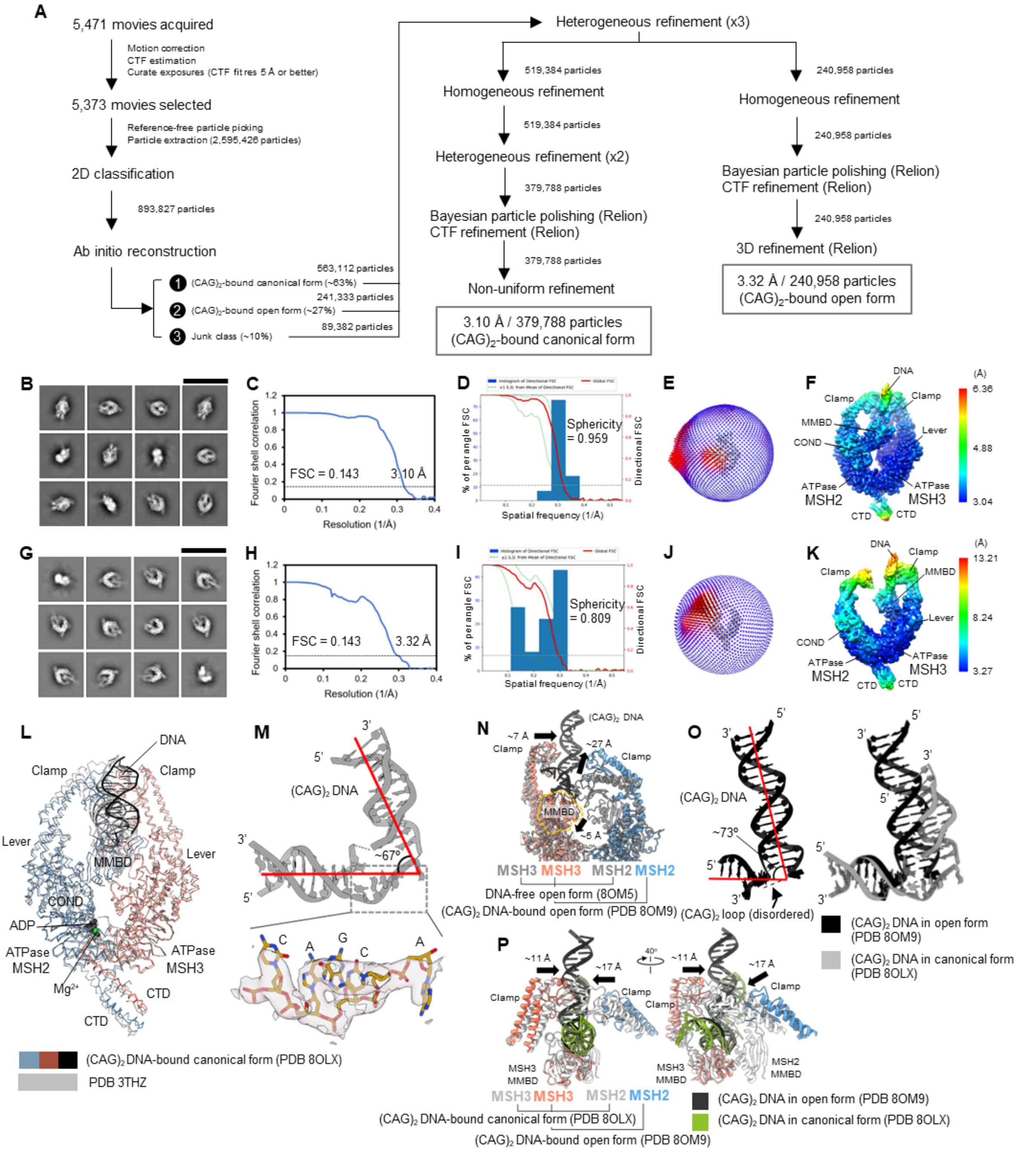

Supplementary Figure S3. Cryo-EM analysis of (CAG)$_2$ DNA-bound canonical and open forms of MutSβ-ADP complex (PDBs 8OLX and 8OM9). (**A**) Summary of the image processing workflow. (**B**) Representative 2D classes of (CAG)$_2$ DNA-bound canonical form of MutSβ (PDB 8OLX). The scale bar in white indicates 293 Å. (**C**) Gold-standard FSC curve for the density map of (CAG)$_2$ DNA-bound canonical form of MutSβ. (**D**) 3D FSC plot for the density map of (CAG)$_2$ DNA-bound canonical form of MutSβ. (**E**) Heat map showing particle orientation distribution of (CAG)$_2$ DNA-bound canonical form. (**F**) Local resolution represented by a heat map on the density contour of (CAG)$_2$ DNA-bound canonical form. (**G**) Representative 2D classes of (CAG)$_2$ DNA-bound open form of MutSβ (PDB 8OM5). The scale bar in white indicates 293 Å. (**H**) Gold-standard FSC curve for the density map of (CAG)$_2$ DNA-bound open form. **(I)** 3D FSC plot for the density map of (CAG)$_2$ DNA-bound open form. (**J**) Heat map showing particle orientation distribution of (CAG)$_2$ DNA-bound open form. (**K**) Local resolution represented by a heat map on the density contour of (CAG)$_2$ DNA-bound open form. (**L**) Ribbon diagram of the mismatch-bound canonical structure of MutSβ, determined by cryo-EM and crystallography (PDB 3THZ), are

shown based on superposition. (**M**) The $(CAG)_2$ DNA structure in the canonical mismatch-bound state of MutSβ, including a close-up view of mis-paired bases overlaid with cryo-EM density (transparent pink) is shown. (**N**) Superposition of the DNA-free open conformation of MutSβ with its $(CAG)_2$ DNA-bound open state. Substantial domain movements are indicated by arrows. (**O**) The $(CAG)_2$ DNA structure in the mismatch-bound open state of MutSβ (left) and its overlay with the same DNA in the canonical mismatch-bound state of MutSβ (right) are shown. (**P**) Two views of superposition of the two $(CAG)_2$ DNA-bound MutSβ cryo-EM structures. The arrows indicate the inward movement of the two clamps in the transition to the canonical high-affinity state.

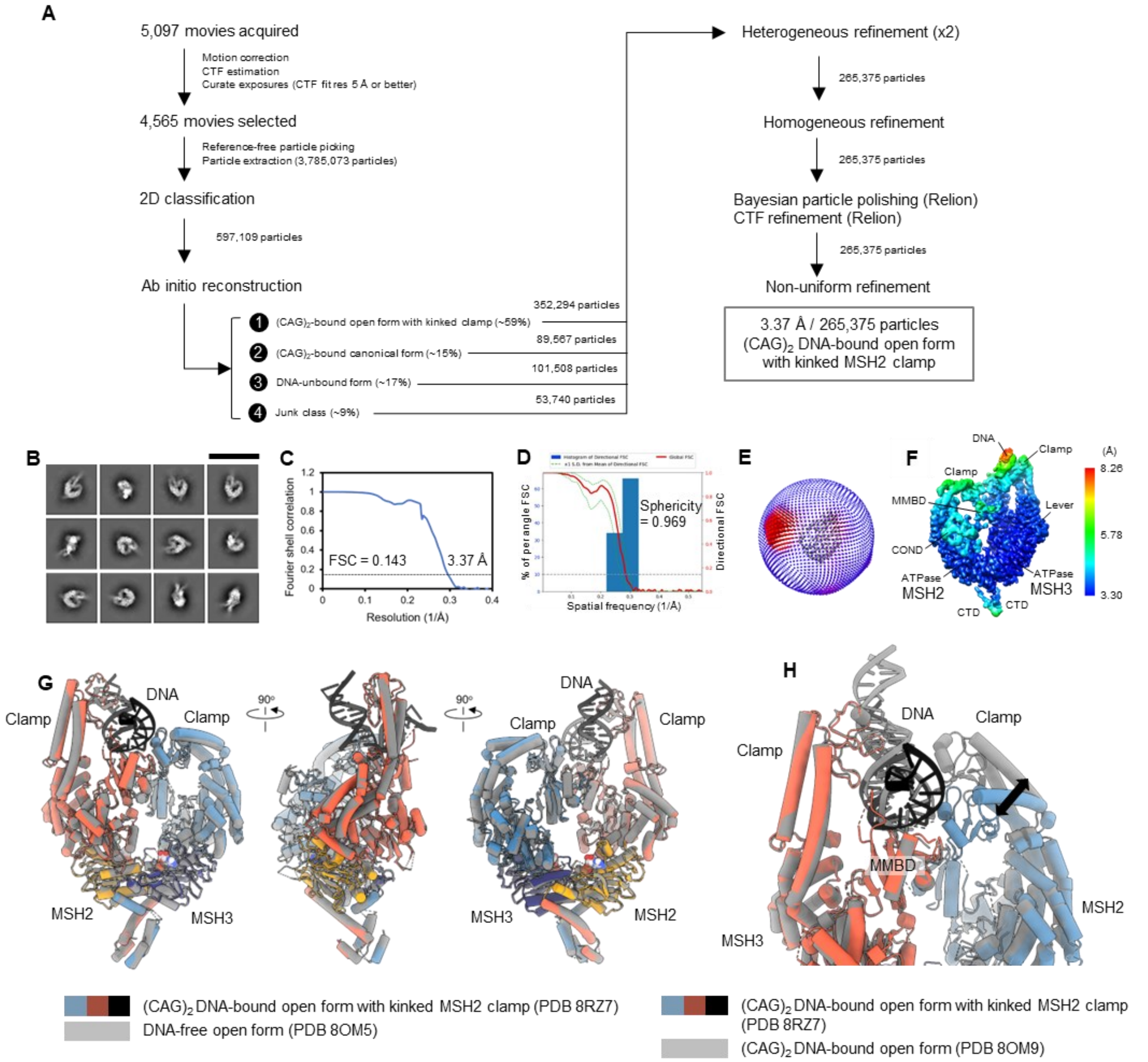

Supplementary Figure S4. Cryo-EM analysis of (CAG)2 DNA-bound open form of MutSβ-ADP complex with kinked MSH2 clamp (PDB 8RZ7). (**A**) Summary of the image processing workflow. (**B**) Representative 2D classes of (CAG)2 DNA-bound open form of MutSβ with kinked MSH2 clamp. The scale bar in white indicates 293 Å. (**C**) Gold-standard FSC curve for the density map. (**D**) 3D FSC plot for the density map. (**E**) Heat showing particle orientation distribution. (**F**) Local resolution represented by a heat map on the density contour. (**G**) Three orthogonal views of the (CAG)2 DNA-bound open form overlaid with the DNA-free open form of MutSβ are shown based on superposition. (**H**) Structural comparison of two cryo-EM structures of MutSβ bound to the same (CAG)2 DNA in two different conformations. The black arrow indicates a substantial movement of the MSH2 clamp.

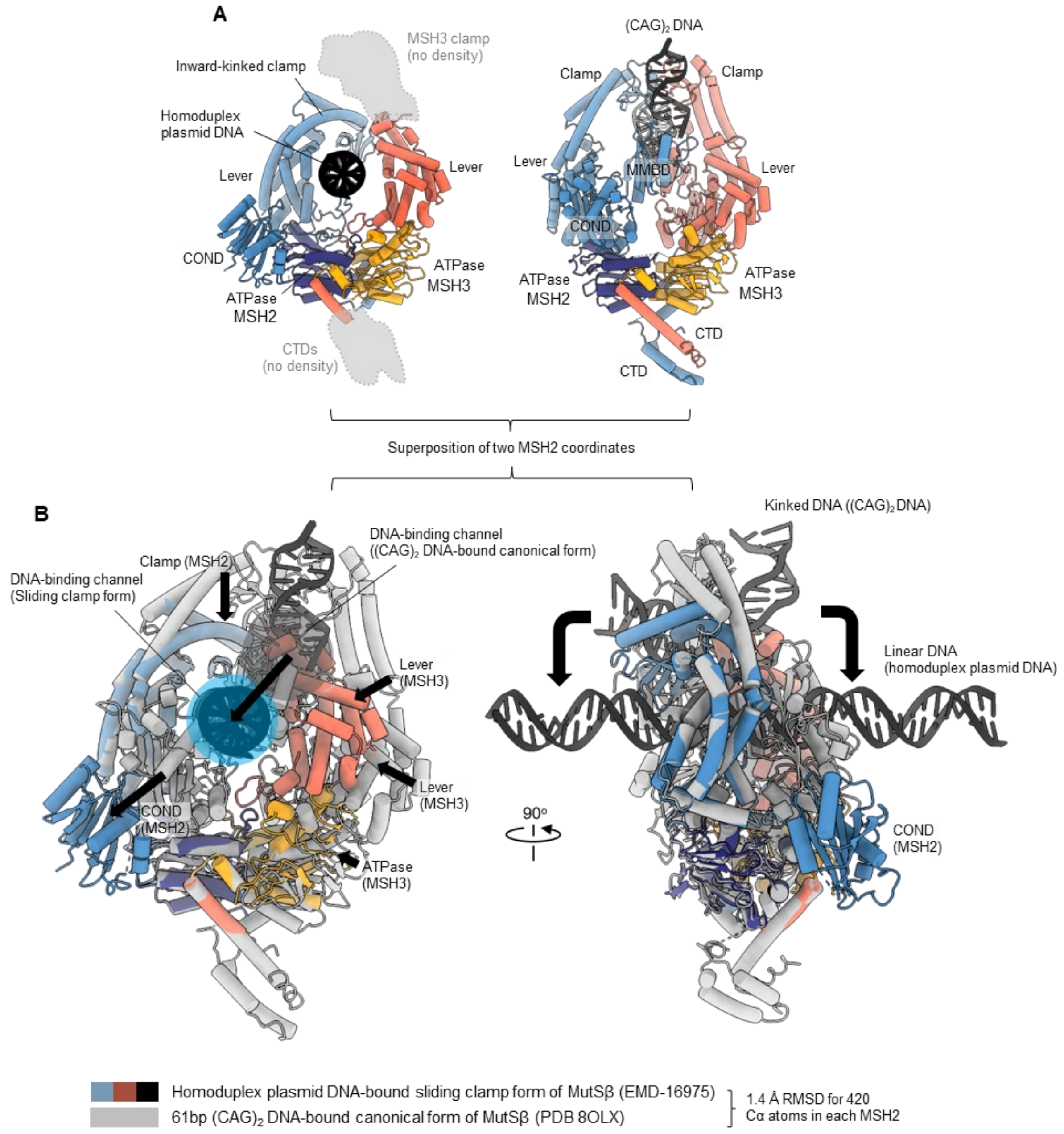

Supplementary Figure S5. Structural comparison of homoduplex plasmid DNA-bound sliding clamp form of MutSβ with its canonical form bound to (CAG)$_2$ DNA. (**A**) Ribbon diagrams of human MutSβ in its DNA-bound form are shown for two different conditions: (1) bound to ~1.8 kb homoduplex plasmid DNA in the presence of ATP (left) and (2) bound to 61 bp (CAG)$_2$ DNA in the absence of added nucleotide (right), based on superposition. (**B**) Two orthogonal views of superimposed cryo-EM structures show conformational differences between two structures. The black arrows indicate domain movements of MSH2, MSH3, and DNA towards the homoduplex plasmid DNA-bound sliding clamp state of Mutβ.

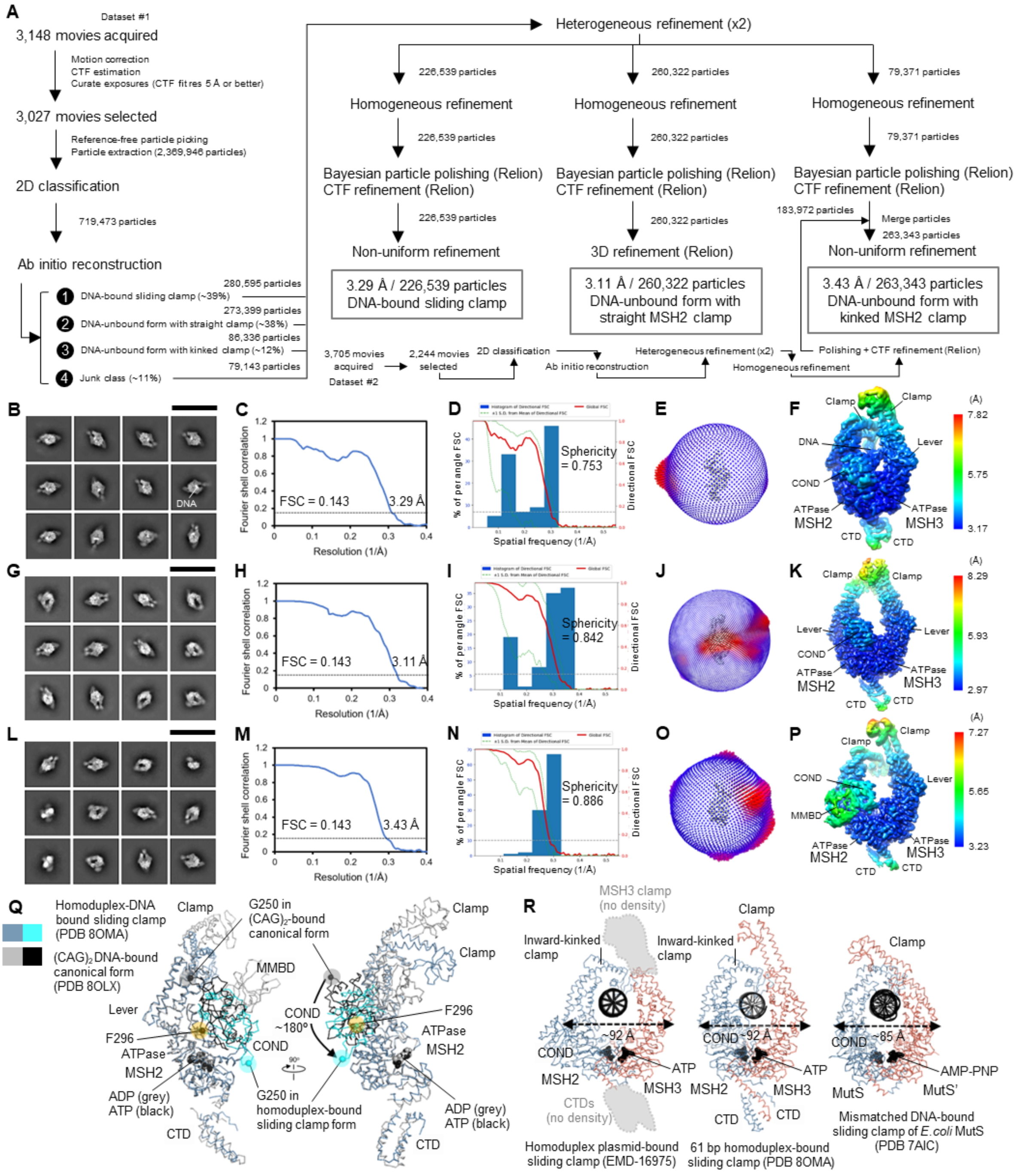

Supplementary Figure S6. Cryo-EM analysis of 61 bp homoduplex DNA-bound sliding clamp (PDB 8OMA) and two DNA-unbound forms of MutSβ-ATP complex (PDBs 8OMO and 8OMQ). (**A**) Summary of the image processing workflow. Representative 2D classes (293 Å scale car in black) (**B**), gold-standard FSC curve for the density map (**C**), 3D FSC42 plot for the density map (**D**), heat map showing particle orientation distribution (**E**), and local resolution represented by a heat map on the density contour (**F**) of 61 bp homoduplex-bound sliding clamp form of MutSβ. The same corresponding images for DNA-unbound form with straight MSH2 clamp (**G-K**) and with kinked MSH2 clamp (**L-P**) are shown in the same order. (**Q**) Ribbon diagrams of the canonical and sliding clamp forms of MutSβ bound to DNA are shown based on superposition. The MSH2 connector rotates around residue F296 compared to the $(CAG)_2$-bound canonical form relative to other domains. The black arrows indicate substantial domain movement of MSH2 connector. (**R**) Ribbon diagrams of human MutSβ and *E. coli* MutS (PDB 7AIC) are shown in their DNA-bound sliding clamp form, based on superposition.

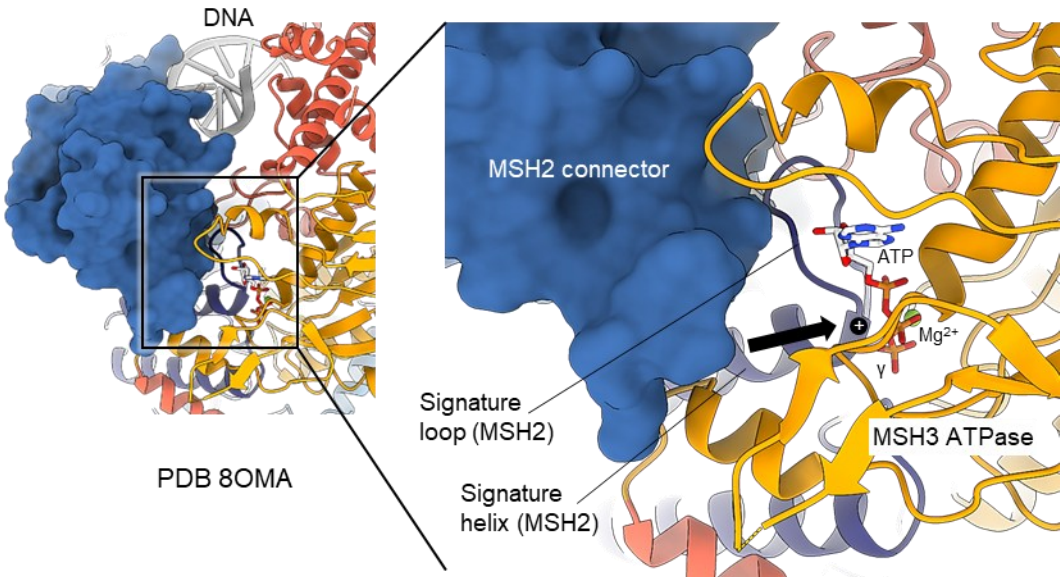

Supplementary Figure S7. DNA-bound sliding clamp conformation of MutSβ (PDB 8OMA), highlighting the nucleotide-binding site of MSH3 with the inward-moved MSH2 connector shown as a surface representation. The arrow indicates the direction of the helix dipole, which contributes to ATP binding stabilization.

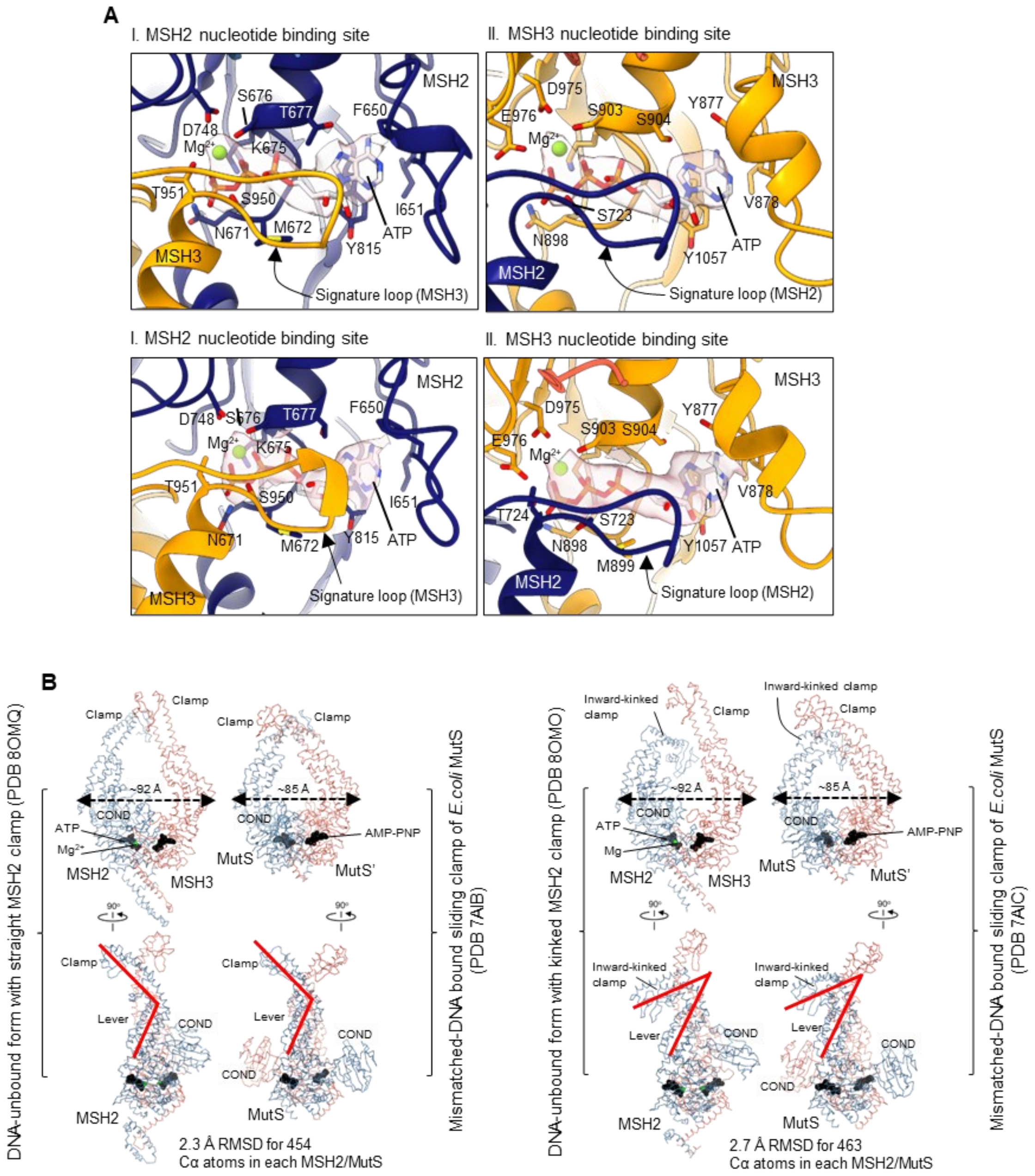

Supplementary Figure S8. Structural analysis of two DNA-unbound forms of MutSβ-ATP complex (PDBs 8OMO and 8OMQ). (**A**) Close-up views of nucleotide binding pockets in the DNA-unbound MutSβ-ATP complexes: straight MSH2 clamp (PDB 8OMQ) (top panels) vs. kinked MSH2 clamp (PDB 8OMO) (bottom panels). Cryo-EM densities for bound ATP-Mg are transparently shown in pink. (**B**) Structural comparison of human MutSβ and *E. coli* MutS homodimer (PDB 7AIB and 7AIC; DNA and MutL are omitted for clarity) are shown in their ATP- or AMP-PNP-bound forms, based on superposition. A sharp kinking of the clamp domain of MSH2 and one subunit of MutS (indicated in red on the right) is shown.

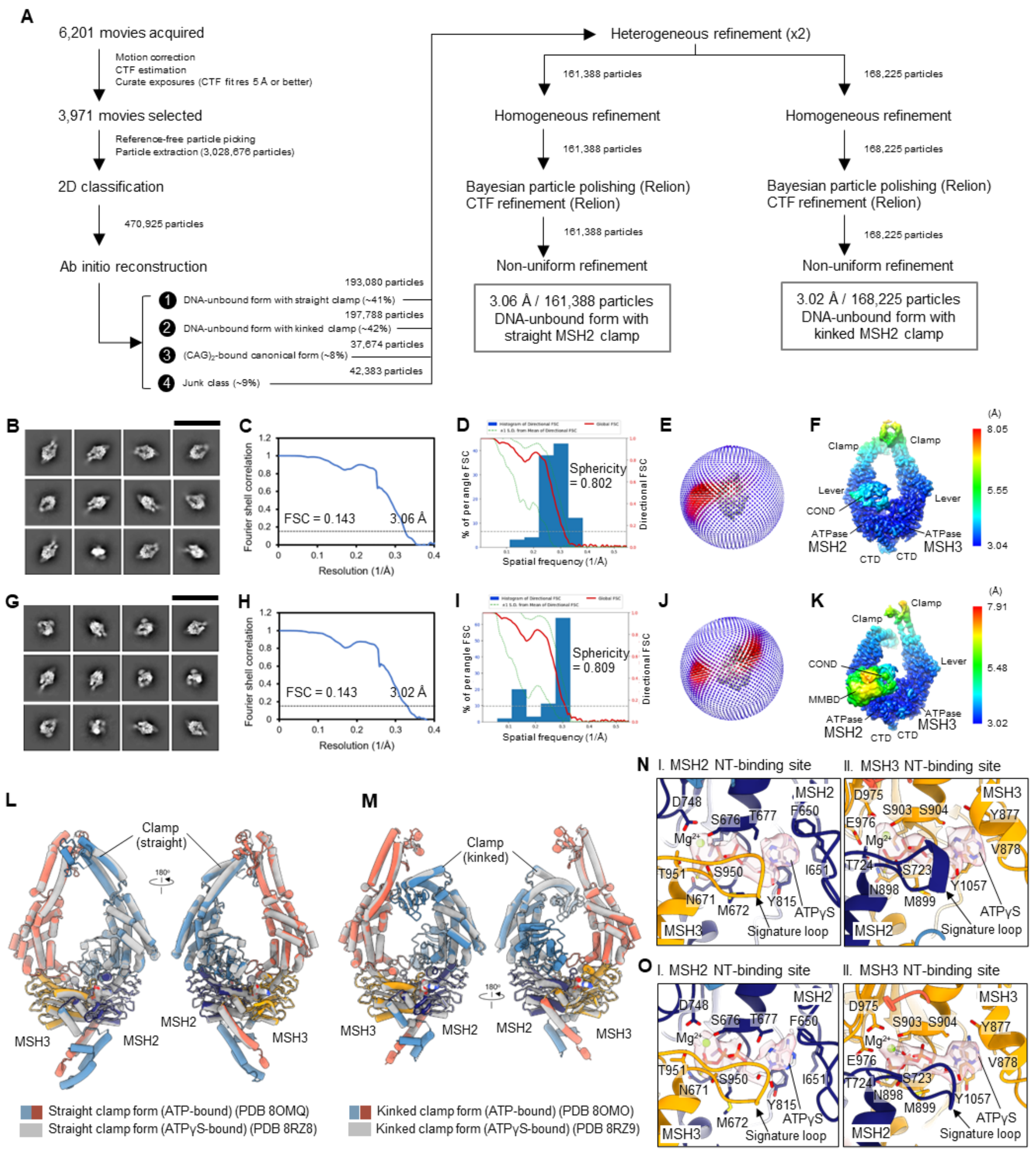

Supplementary Figure S9. Cryo-EM analysis of DNA-unbound MutSβ-ATPγS complex with straight (PDB 8RZ8) or kinked MSH2 clamp (PDB 8RZ9). (**A**) Summary of the image processing workflow. Representative 2D classes (293 Å scale bar in black) (**B**), gold-standard FSC curve for the density map (**C**), 3D FSC plot for the density map (**D**), heat map showing particle orientation distribution (**E**), and heat map showing particle orientation distribution (**F**) of DNA-unbound MutSβ-ATPγS complex with straight MSH2 clamp. The corresponding images for the DNA-unbound MutSβ-ATPγS complex with kinked MSH2 clamp are shown in the same order (**G-K**). (**L**) Structural comparison of DNA-unbound forms of MutSβ bound to ATP or ATPγS with straight MSH2 clamp is shown based on superposition. (**M**) Structural comparison of DNA-unbound forms of MutSβ bound to ATP or ATPγS with kinked MSH2 clamp is shown based on superposition. Close-up views of nucleotide binding pockets in the DNA-unbound MutSβ-ATPγS complexes: straight MSH2 clamp (**O**) vs. kinked MSH2 clamp (**P**). Cryo-EM densities for bound ATPγS-Mg are transparently shown in pink.

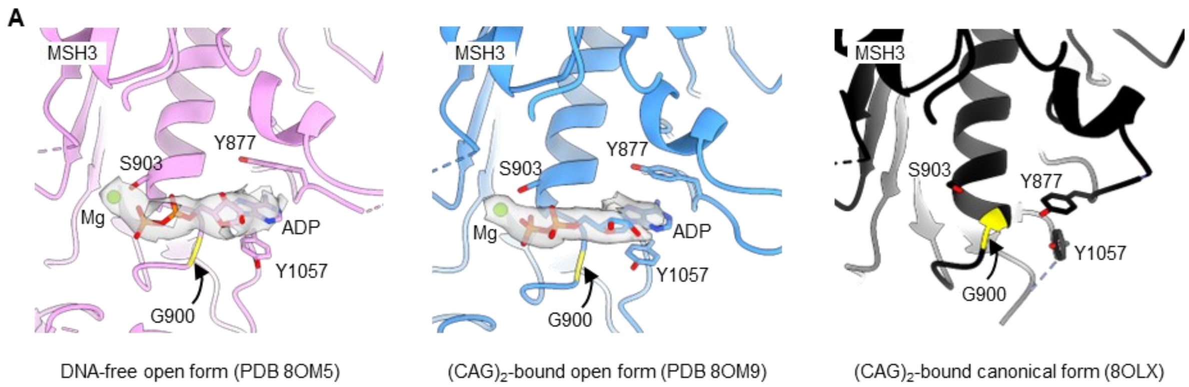

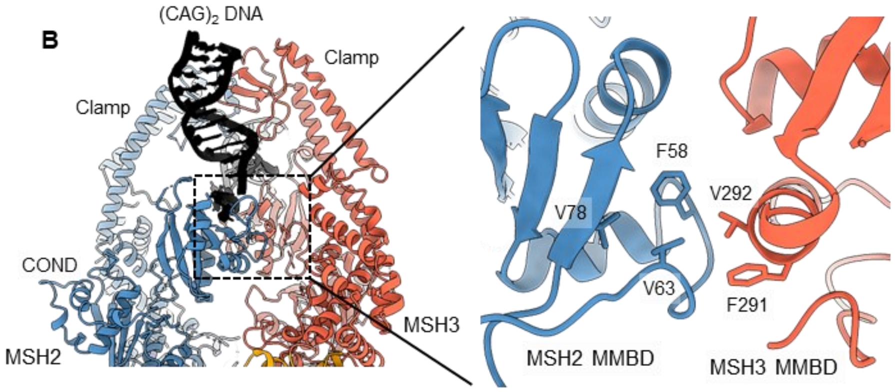

Supplementary Figure S10. Structural analysis of different conformational states of human MutSβ. (**A**) Ribbon diagrams of the MSH3 site in the DNA-free open form of MutSβ (left) and its $(CAG)_2$ DNA-bound form (middle and right) are shown, based on structural superposition. The MSH3 G900 residue in the Walker A motif is highlighted in yellow. In the canonical form of MutSβ complexed with $(CAG)_2$ DNA, the MSH3 Y877 residue occupies the adenine-binding site, leading to a lack of nucleotide at the MSH3 nucleotide-binding pocket. Cryo-EM densities for bound ADP-Mg are transparently shown in gray. (**B**) Hydrophobic interface between MSH2 and MSH3 MMBDs in the $(CAG)_2$ DNA-bound canonical form of MutSβ (PDB 8OLX).

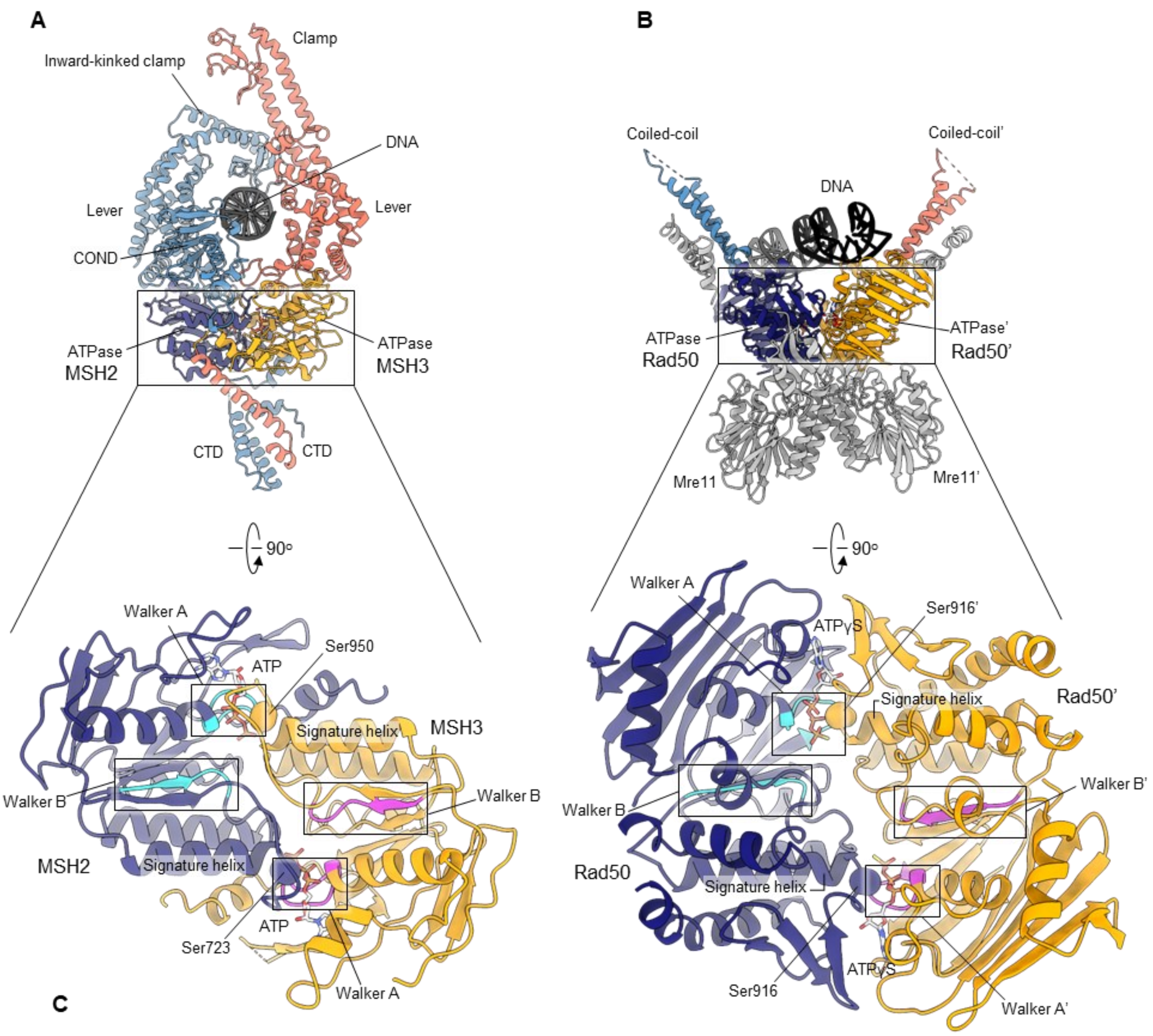

C

Walker A motifs

```
Human MSH2            669 GPNMGGKS 676
Human MSH3            896 GPNMGGKS 903
Methanococcus Rad50    32 GENGSGKS 39
```

Walker B motifs

```
Human MSH2            744 LIIIDE 749
Human MSH3            971 LVILDE 976
Methanococcus Rad50   942 CIILDE 947
```

Supplementary Figure S11. Structural comparison of the DNA-bound MutSβ sliding clamp with bound ATP (PDB 8OMA) and the DNA-bound Mre11-Rad50 complex with bound ATPγS (PDB 5DNY). (**A**) Two orthogonal views of the DNA-bound MutSβ sliding clamp with bound ATP, shown as ribbon representations. The ATPase domains of MSH2 and MSH3 are magnified to display key structural elements, including Walker A and B motifs, bound ATP, and serine residues that contribute to ATP stabilization at the MSH2 and MSH3 sites. (**B**) Two orthogonal views of the DNA-bound Rad50 homodimer with bound ATPγS, shown as ribbon representations. The ATPase domains are magnified to highlight key structural elements, including Walker A and B motifs, bound ATPγS, and serine residues involved in ATPγS stabilization at both Rad50 sites. (**C**) Amino acid sequence alignment of the Walker A and B motifs from human MSH2, human MSH3, and Methanococcus Rad50. Conserved residues are highlighted in yellow.

Supplementary Table S1. Sequences of dsDNA substrates used in this study.

dsDNA substrate	Strand	Sequence	Modification
**61 bp homoduplex, end-capped**	Forward	5‘-<br>CTGAAGCTTAGCTTAGGATCATCGAGGATCAGCTCGGTGCAATTCA<br>GCGGTACCCAATTC-3‘	5‘ Biotin
	Reverse	5‘-<br>GAATTGGGTACCGCTGAATTGCACCGAGCTGATCCTCGATGATCCT<br>AAGCTAAGCTTCAG-3‘	5‘ Biotin
**61 bp $(CAG)_2$, end-capped**	Forward	5‘-<br>CTGAAGCTTAGCTTAGGATCATCGAGGATCCAGCAGAGCTCGGTG<br>CAATTCAGCGGTACCCAATTC-3‘	5‘ Biotin
	Reverse	5‘-<br>GAATTGGGTACCGCTGAATTGCACCGAGCTGATCCTCGATGATCCT<br>AAGCTAAGCTTCAG-3‘	5‘ Biotin
**61 bp $(CAG)_2$, open-ended**	Forward	5‘-<br>CTGAAGCTTAGCTTAGGATCATCGAGGATCCAGCAGAGCTCGGTG<br>CAATTCAGCGGTACCCAATTC-3‘	-
	Reverse	5‘-<br>GAATTGGGTACCGCTGAATTGCACCGAGCTGATCCTCGATGATCCT<br>AAGCTAAGCTTCAG-3‘	-
**70 bp homoduplex, end-capped**	Forward	5‘-<br>CACACGTTCCGAGATATCCTAGCAAGTGATCGTCTATG**T**AGCTCAA<br>GAGTTCGACTCAGCCTACACCGAG-3'	5‘ Digoxigenin, Cy5 (T)
	Reverse	5‘-<br>CTCGGTGTAGGCTGAGTCGAACTCTTGAGCTACATAGACGATCACT<br>TGC**T**AGGATATCTCGGAACGTGTG-3'	5‘ Digoxigenin, Cy3 (T)
**70 bp CAG, end-capped**	Forward	5‘-<br>CACACGTTCCGAGATATCCTAGCAAGTGAT**CAG**CGTCTATG**T**AGCT<br>CAAGAGTTCGACTCAGCCTACACCGAG-3'	5‘ Digoxigenin, Cy5 (T)
	Reverse	5'-<br>CTCGGTGTAGGCTGAGTCGAACTCTTGAGCTACATAGACGATCACT<br>TGC**T**AGGATATCTCGGAACGTGTG-3'	5‘ Digoxigenin, Cy3 (T)
**70 bp $(CAG)_2$, end-capped**	Forward	5‘-<br>CACACGTTCCGAGATATCCTAGCAAGTGAT**CAGCAG**CGTCTATG**T**A<br>GCTCAAGAGTTCGACTCAGCCTACACCGAG-3‘	5‘ Digoxigenin, Cy5 (T)
	Reverse	5‘-<br>CTCGGTGTAGGCTGAGTCGAACTCTTGAGCTACATAGACGATCACT<br>TGC**T**AGGATATCTCGGAACGTGTG-3‘	5‘ Digoxigenin, Cy3 (T)
**70 bp $(CAG)_4$, end-capped**	Forward	5‘-<br>CACACGTTCCGAGATATCCTAGCAAGTGAT**CAGCAGCAGCAG**CGT<br>CTATG**T**AGCTCAAGAGTTCGACTCAGCCTACACCGAG-3'	5‘ Digoxigenin, Cy5 (T)
	Reverse	5'-<br>CTCGGTGTAGGCTGAGTCGAACTCTTGAGCTACATAGACGATCACT<br>TGC**T**AGGATATCTCGGAACGTGTG-3'	5‘ Digoxigenin, Cy3 (T)

Supplementary reference:

Tan, Y.Z., Baldwin, P.R., Davis, J.H., Williamson, J.R., Potter, C.S., Carragher, B. and Lyumkis, D. (2017) Addressing preferred specimen orientation in single-particle cryo-EM through tilting. *Nat. Methods*, **14**, 793–796.
